# Morphological variation under domestication: how variable are chickens?

**DOI:** 10.1098/rsos.180993

**Published:** 2018-08-08

**Authors:** Madlen Stange, Daniel Núñez-León, Marcelo R. Sánchez-Villagra, Per Jensen, Laura A. B. Wilson

**Affiliations:** 1 Paläontologisches Institut und Museum, Universität Zürich, Karl Schmid-Strasse 4, 8006 Zürich, Switzerland; 2 AVIAN Behavioural Genomics and Physiology Group, IFM Biology, Linköping University, 581 83 Linköping, Sweden; 3 Palaeontology, Geobiology and Earth Archives Research Centre, School of Biological, Earth and Environmental Sciences, University of New South Wales, Sydney, New South Wales 2052, Australia

**Keywords:** skull, modularity, morphological integration, neural crest, fowl, morphological diversity

## Abstract

The process of domestication has long fascinated evolutionary biologists, yielding insights into the rapidity with which selection can alter behaviour and morphology. Previous studies on dogs, cattle and pigeons have demonstrated that domesticated forms show greater magnitudes of morphological variation than their wild ancestors. Here, we quantify variation in skull morphology, modularity and integration in chickens and compare those to the wild fowl using three-dimensional geometric morphometrics and multivariate statistics. Similar to other domesticated species, chickens exhibit a greater magnitude of variation in shape compared with their ancestors. The most variable part of the chicken skull is the cranial vault, being formed by dermal and neural crest-derived bones, its form possibly related to brain shape variation in chickens, especially in crested breeds. Neural crest-derived portions of the skull exhibit a higher amount of variation. Further, we find that the chicken skull is strongly integrated, confirming previous studies in birds, in contrast to the presence of modularity and decreased integration in mammals.

## 1. Introduction

The diversity of domesticated fowl (*Gallus gallus*) sparked the interest of Charles Darwin, leading him to dedicate an entire chapter to them in *The variation of animals and plants under domestication* [1]. Selective breeding has produced different kinds of chickens: some are used for meat (broilers, with extreme growth rates [2]), for egg-laying or for ornamental purposes [3]. As noted by Darwin [1], domestication greatly affects morphological diversity—disparity [4]—particularly evidenced in skulls, a recognized marker of disparity in birds [5].

For decades, chickens have been used as a model organism for the study of vertebrate development [6], but their morphological variability has never been quantified and the skull anatomy of breeds in comparison to fowl has never been described. We explored skull shape of wild and domesticated fowl. Quantitative analyses show that the variability of domesticated forms is much larger than that of wild forms, in dogs [7], pigeons [4], cattle [8] and horses [9]. The association of traits into modules (=modularity) and low magnitudes of trait intercorrelation (=integration) have together been hypothesized to generate morphological variation [10]. Empirical data for mammals, however, show that patterns of integration are related to the magnitude of size variation in a clade and remain stable on a macro-scale [11,12]. Whether the same pattern is also true for domesticated animals, considered by some as a case of rapid evolution [13], has barely been tested.

We address several questions (Q) relating to the magnitude and patterning of cranial shape variation: (Q1) Do domesticated forms exhibit greater morphological variability (as measured by Procrustes variance (PV)) than do wild forms? (Q2) Is there a significant effect of size on shape variation? (Q3) Is there evidence for modularity or integration between mesodermal and neural crest-derived portions of the chicken skull? (Q4) Is the magnitude of variation in neural crest- versus mesoderm-derived parts similar?

## 2. Material and methods

### 2.1. Sampling

Chicken breeds are represented by numerous varieties, and many of them have been interbred to obtain traits such as a high egg-laying rate [3,14]. In contrast to dogs, for which breeds are standardized regionally and internationally, there is no worldwide classification system for chicken breeds and standards from different regions do not match exactly in their definition for many breeds [15,16]. The sampling in our study does not cover the diversity of breeds at large [17,18] but with its coverage, it represents much of skull variation. All in all, the sample contains specimens of the main wild fowl form (red junglefowl, RJF) from which chickens derive [19], skulls of chicken breeds that were bred for egg-, or meat-production, as well as for ornamental or fight purposes, from small (true bantams) to large (meat-type) breeds, from common (egg-type breeds) to peculiar breeds (Polish). Our sampling includes the controversial Araucana [20], three rare Swiss breeds including a crested breed; Polish chickens, characterized by a protuberance of the skull, a common phenomenon in crested breeds; and the rare Burmese bantam, specimens of the latter two collected by Charles Darwin himself. In total, we investigated skulls of 62 *Gallus* spp., comprising 21 wild fowl and 41 chickens (table 1).

**Table 1.** List of analysed specimens, breeds, varieties and their use. PIMUZ, Paläontologisches Museum Universität Zürich; SAPM, Staatssammlung für Anthropologie und Paläoanatomie München; NHMUK, Natural History Museum United Kingdom, Bird Collection, Tring; NHMUK*, Natural History Museum United Kingdom, Bird Collection, Tring, Darwin collection; PCGB, Poultry Club of Great Britain; APA, American Poultry Association. n/a, information not available or breed not classified. CB, crossbreed of red junglefowl (RJF) and Mrs Taylor (Mrs T) wild-type RJF [22]. Breed names in quotes refer to their original museum labels but cannot be allocated to recognized breeds. *Gallus gallus* (red junglefowl) and *Gallus sonneratii* (grey junglefowl) have been shown to have contributed to the genomic and morphological variation of chickens [23].

museum	catalogue no.	breed	classification for disparity and form space	Storey's grouping by use [3]
NHMUK	S/1989.19.1	*Gallus gallus jabouillei*	fowl	outgroup
NHMUK	1945.1.1	*Gallus lafayettii*	fowl	outgroup
NHMUK	1864.11.27.13	*Gallus sonneratii*	fowl	outgroup
NHMUK	S/1975.105.13	*Gallus sonneratii*	fowl	outgroup
NHMUK	S/1985.41.2	*Gallus sonneratii*	fowl	outgroup
SAPM	173	*Gallus gallus*	fowl	outgroup
NHMUK	S/1999.43.40	*Gallus gallus*	fowl	outgroup
PIMUZ	PIM_RJF_1_f	*Gallus gallus*	fowl	outgroup
PIMUZ	PIM_RJF_2_f	*Gallus gallus*	fowl	outgroup
PIMUZ	PIM_RJF_3_f	*Gallus gallus*	fowl	outgroup
PIMUZ	PIM_RJF_4_f	*Gallus gallus*	fowl	outgroup
PIMUZ	PIM_RJF_5_f	*Gallus gallus*	fowl	outgroup
PIMUZ	PIM_RJF_6_m	*Gallus gallus*	fowl	outgroup
PIMUZ	PIM_RJF_7_m	*Gallus gallus*	fowl	outgroup
PIMUZ	PIM_RJF_8_m	*Gallus gallus*	fowl	outgroup
PIMUZ	PIM_RJF_9_m	*Gallus gallus*	fowl	outgroup
PIMUZ	PIM_RJF_10_m	*Gallus gallus*	fowl	outgroup
NHMUK	S/1999.43.42	CB	fowl	outgroup
NHMUK	S/1999.43.55	CB	fowl	outgroup
NHMUK	S/2009.10.1	CB	fowl	outgroup
NHMUK	S/1999.43.35	CB (75% RJF x 25% Mrs T (‘Gollum’) [21])	fowl	outgroup
NHMUK*	1868.2.19.53	‘African cock’	chicken	n/a
PIMUZ	PIM_Bh_1	Appenzeller Barthuhn	chicken	egg
PIMUZ	PIM_Bh_2	Appenzeller Barthuhn	chicken	egg
PIMUZ	PIM_Bh_3	Appenzeller Barthuhn	chicken	egg
PIMUZ	PIM_Bh_4	Appenzeller Barthuhn	chicken	egg
PIMUZ	PIM_Sph_1	Appenzeller Spitzhaubenhuhn	chicken	egg
PIMUZ	PIM_Sph_2	Appenzeller Spitzhaubenhuhn	chicken	egg
PIMUZ	PIM_Sph_3	Appenzeller Spitzhaubenhuhn	chicken	egg
PIMUZ	PIM_Sph_4	Appenzeller Spitzhaubenhuhn	chicken	egg
PIMUZ	PIM_Sph_5	Appenzeller Spitzhaubenhuhn	chicken	egg
PIMUZ	PIM_Sph_6	Appenzeller Spitzhaubenhuhn	chicken	egg
PIMUZ	PIM_Sph_7	Appenzeller Spitzhaubenhuhn	chicken	egg
SAPM	115	Araucana (crested)	chicken	egg
SAPM	84	Araucana	chicken	egg
SAPM	86	Araucana	chicken	egg
SAPM	87	Araucana	chicken	egg
NHMUK	S/1851.12.3.10	‘Bantam’	chicken	n/a
NHMUK*	1868.2.19.74	Burmese (bantam)	chicken	ornamental
SAPM	60	Cochin	chicken	ornamental
NHMUK*	1868.2.19.65	‘game hen’	chicken	ornamental/fight
NHMUK*	S/1868.2.19.57	Hamburgh, golden penciled	chicken	egg
SAPM	113	‘Haushuhn’	chicken	n/a
SAPM	7	leghorn	chicken	egg
SAPM	63	‘Holländisches Huhn’^a^	chicken	n/a
NHMUK	S/1999.43.36	leghorn	chicken	egg
NHMUK	S/1999.43.56	leghorn	chicken	egg
NHMUK	S/1952.3.51	Malay/Kulm	chicken	meat/fight
NHMUK	1900.7.6.20	*Gallus gallus* dom.	chicken	n/a
NHMUK	1859.9.6.347	*Gallus gallus* dom.	chicken	n/a
NHMUK	S/2001.50.116	*Gallus gallus* dom.	chicken	n/a
PIMUZ	ZH	*Gallus gallus* dom.	chicken	n/a
SAPM	64	Pekin (bantam)	chicken	ornamental
NHMUK*	1868.2.19.61	Polish, gold spangle	chicken	ornamental
NHMUK*	S/1952.2.114	Polish, white	chicken	ornamental
NHMUK*	1868.2.19.58	Rumpless Game	chicken	ornamental/fight
PIMUZ	PIM_Sh_1	Schweizer Huhn	chicken	meat
PIMUZ	PIM_Sh_2	Schweizer Huhn	chicken	meat
PIMUZ	PIM_Sh_3	Schweizer Huhn	chicken	meat
PIMUZ	PIM_Sh_4	Schweizer Huhn	chicken	meat
NHMUK*	1868.2.19.67	‘Spanish cock’	chicken	egg
NHMUK*	S/1952.2.105	‘Spanish cock’	chicken	egg

### 2.2. Data acquisition and generalized procrustes superimposition

**Figure 1.**
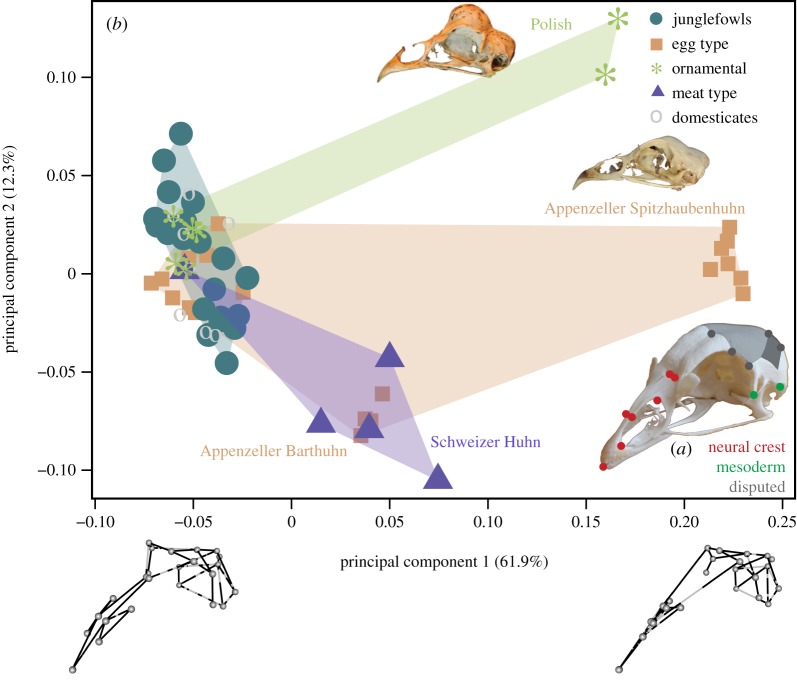
(*a*) Image of *Gallus gallus* showing landmarks captured in this study. Dot colour: neural crest (red), mesoderm (green), disputed origin (grey), as described in table 3. The posterior part of the frontal and parietal are highlighted in shades of grey, respectively (see §2.5 for explanation). (*b*) PC1–PC2-scatterplot of all analysed specimens (see electronic supplementary material, table S1); Appenzeller Spitzhaubenhuhn (middle) and Polish (top) represent crested breeds with unusual upper beak anatomy. Line graphs of extremal shapes are shown below the first PC.

We used three-dimensional (3D) geometric morphometrics to visualize and test for differences in skull shape and occupation of shape space between fowl and chickens. Each skull was digitized in three-dimensional space using a MicroScribe^®^ MLX6 to capture 24 landmarks (figure 1*a*, table 2). As a first step, digitizing error was assessed by Procrustes ANOVA and yielded no significant differences among replicates (*F* = 0.7927, *p* = 0.671). Next, generalized Procrustes superimposition [25] accounting for bilateral symmetry [26,27] was performed with the R package geomorph v.3.0.4 [28] in the R v.3.3.3 [29] environment, to remove the effects of size, orientation and position, resulting in symmetric and asymmetric components. The Procrustes ANOVA performed with the bilat.symmetry function showed an effect of fluctuating asymmetry (sum of squares (SS) = 0.17) and directional asymmetry (SS = 0.07) on shape, both much smaller than the effect of inter-specimen differences (SS = 1.25).

**Table 2.** Description of the positioning of landmarks on fowl and chicken skulls used for geometric morphometric analysis.

landmark no.	description	type of landmark after [24]
1	distal-most tip of upper jaw in the midline	2
2,3	anterior-ventral tip of the nasal pit	2
4,5	anterior tip of the dorsal nasal processes	2
6,7	medial tip of the posterior part of the nasal pit	2
8,9	posterior-most tip of dorsal premaxilla process	2
10,11	‘point where frontal goes to ventral’	2
12	middle point between 10 and 11 (crest)	3
13,14	posterior end of frontal	2
15	midline junction between frontal and parietal	1
16	midline junction between parietal and supraoccipital	1
17	posterior-most tip of cranium	2
18,19	tip of postorbital process	2
20,21	medial tip of ‘squamosal curvature’	2
22,23	lateral tip of the basitemporal	2
24	middle point of the dorsal foramen magnum	2

**Table 3.** Alternative assignments of landmarks to modules following Couly [40] and Noden [42–44] for analyses of modularity, integration and magnitude of variation. In this study, we use the different hypotheses of Couly *et al*. [40] and Noden [42–44] as a basis to test for modular structure and integration in the chicken skull while taking into account the changing hypothesized boundaries of mesodermal and neural crest-derived portions. The landmarks are located at hypothesized boundaries, which are highlighted in grey in figure 1*a*.

	module	*H*_Couly_	*H*_Noden_
alternative 1	neural crest	1–16, 20, 21	1–9, 20, 21
	mesoderm	17–19, 22–24	10–19, 22–24
alternative 2	neural crest	1–15	1–12
	mesoderm	16–24	13–19, 20–24
alternative 3	neural crest	1–15, 20, 21	1–12, 20, 21
	mesoderm	16–19, 22–24	13–19, 22–24

### 2.3. Visual and statistical analysis of skull shape space occupation of fowl and chickens

Principal component analysis (PCA) was used to explore and visualize morphospace occupation along the major axes of variance for (i) all fowl and chickens and (ii) without crested breeds (Polish and Appenzeller Spitzhaubenhuhn), which exhibit a skeletal peculiarity by missing the characteristic nasals/premaxillar meeting in birds and, therefore, dominated the variance along principal component 1 (PC1). To test for differences in shape space dispersion between fowl and chicken, we applied an analogue of the Levene's test, an analysis of multivariate homogeneity of group dispersions [30,31]. To compare the mean shapes of chickens and fowl, the mean shape configuration was computed for RJF and CB together as fowl, as well as for the non-crested chicken breeds as chickens together.

### 2.4. Analysis of the impact of size on shape (allometry) between fowl and chickensand within chickens

**Figure 2.**
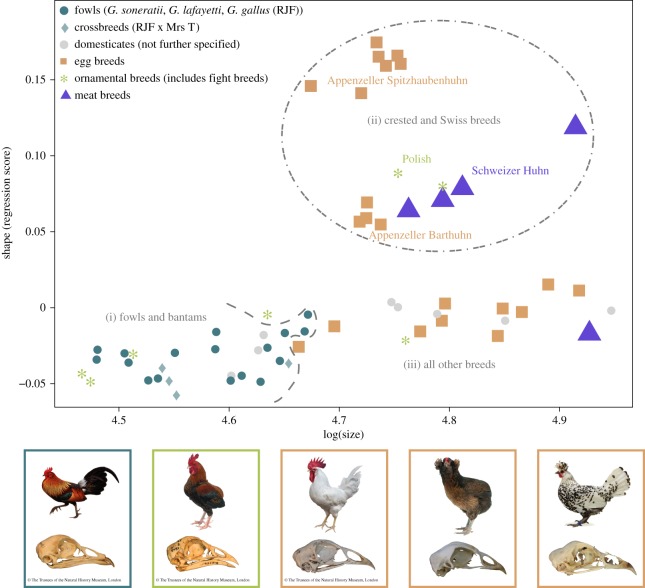
Multivariate regression of shape on log centroid size (log (Size)). Images illustrate exemplarily variation among junglefowl (left); ornamental breeds, here a fighting breed (second left); egg-laying breeds, Leghorn (middle), South-American Araucana (second right) and Appenzeller Spitzhaubenhuhn. Size and shape are weakly correlated in fowl and chickens (6.6%, *p* = 0.021) and both exhibit different mean shapes. Trajectory length and slope were compared between groups (i, ii and iii).

To test whether size and shape covary (allometry), Procrustes coordinates (representing shape) were regressed on log centroid sizes by using the procD.allometry function. This function was applied to the entire sample and to fowl and chickens, separately. The relationship of shape and log centroid size for the entire sample was plotted for visual inspection based on the regression scores [21] which is in this case identical to the common allometric component [32]. To test for covariation of shape and size together with a grouping factor (i.e. fowl versus chickens; fowl and bantams (i) versus Swiss and Polish breeds (ii); and Swiss and Polish breeds (ii) versus all other breeds and fowl (i + iii); figure 2 for explanation of grouping factors), the procD.lm function was applied. When the interaction term of size and the grouping factor was significant, a homogeneity of slopes (HOS) test was performed in advanced.procD.lm to test for differences in slope properties between the groups. The HOS performs pairwise comparisons of the slope angles (direction of shape change with size) and slope vector lengths (amount of shape change with size) [33–35]. In the case where the pairwise comparisons of slope angle do not reveal a significant difference, the null hypothesis of common slopes (with variable intercepts) cannot be rejected. The advanced.procD.lm function was used to perform a test of least square (LS) means, to assess whether there was a shift in intercept along the *Y*-axis (shape) between the two groups. In all analyses, significance was evaluated with a residual randomization permutation procedure with 1000 iterations [34,36–38].

### 2.5. Analysis of modularity and integration within chickens and magnitude of variation in neural crest- and mesoderm-derived parts of the skull

In birds, there is disagreement about the origin of some skull elements [39], in particular, the neural crest/mesoderm boundary between the frontal and parietal. The frontal has been found to be derived exclusively from neural crest by Couly *et al.* [40], or to be of mixed origin [41] being formed from neural crest and mesodermal cells as identified by Noden [42–44]. The parietal has been found to be of mesodermal origin by different authors [41,44,45] or of neural crest origin by Couly *et al.* [40]. We thus examine partitions of the skull following alternative hypotheses [40,44] (table 3). We denote the contrasting hypotheses as *Noden* versus *Couly*.

The alternative hypotheses of modularity (figure 1*a*) were tested with the null hypothesis of no modular structure based on the covariance ratio (CR), using the modularity.test function in geomorph [46]. We tested all possible combinations with changing membership of landmarks that lie on the boundary of mesodermal to neural crest-derived bones (summarized in table 3). A CR value smaller than one expresses low covariation among modules supporting modular structure, CR = 1 is found among random sets of landmarks, and CR > 1 indicates that the covariation among landmarks of the different modules is larger within than between modules. Significance was quantified by random assignment of landmarks to modules in 1000 permutations. Subsequently, the strength of covariation (integration) among the hypothesized modules was tested using a two-block partial least-squares analysis [33,34,47].

The magnitude of variation (a) within the hypothesized partitions (table 3) of mesodermal and ectodermal origin of the chicken skull was assessed using the morphol.disparity function in geomorph [48] by calculating overall PV and PV for each hypothesized module separately and by reporting these values in relation to the amount of landmarks present in that partition, as well as standardized by the number of landmarks per partition (i.e. PV/# LM) (see [49] for similar approach). Further, to estimate (b) which landmarks were most variable we calculated variation using two approaches. First, we calculated the maximum Euclidean distance in PAST2 [50] across all specimens from a mean set of *x-*, *y-*, *z-*coordinates, performed for each landmark separately. Second, we used the morphol.disparity function in geomorph to quantify PV for each landmark separately. PV is calculated as the sum of the diagonal elements in the group covariance matrix divided by the number of elements in the group.

## 3. Results

### 3.1. Differentiation of fowl and chickens in skull shape space

The PCA of all wild and domesticated specimens (figure 1*b*) highlights the osteological difference of the crested breeds, the Polish and the Appenzeller Spitzhaubenhuhn. The Polish exhibits a cranial protuberance, and both breeds are characterized by very short premaxillary and nasal processes, which leave a ‘gap' in the upper beak. Exclusion of the crested breeds from PCA (electronic supplementary material, figure S1) shows that all breeds but the Swiss fall into the same area of PC1–PC2 space as fowl. Fowl, as compared with chickens, are characterized by a narrower skull, less downward curved premaxilla, flatter cranial vault and a shorter but more globular braincase. The analysis of multivariate homogeneity of group dispersions [30] revealed significant differences between fowls and chickens (*F* = 13.73, *p* = 0.0005).

### 3.2. Testing for divergent patterns of allometry between fowl and chickens

Testing for allometry by regression of skull shape (Procrustes coordinates) on log centroid size (figure 2) for the entire sample (chickens and fowl) shows a weak effect of size on shape (*R*^2^ = 6.6%, *p* = 0.021). Procrustes ANOVA results indicate that the mean shapes of fowl and chickens (*F* = 4.2, *p* = 0.013) but not their allometries (*F* = 0.47, *p* = 0.657) differ. Individual tests for allometry within fowl and chickens are non-significant (*F* = 1.37, *p* = 0.23; *F* = 1.04, *p* = 0.34, respectively). Within chickens a second cohort that separates from the common trajectory with fowl is found in the upper right part of the scatterplot (figure 2) and consists of the Polish and rare Swiss breeds. Fowl and bantams (i) and all remaining except Swiss and Polish breeds (iii) share a common allometry (*F* = 0.78, *p* = 0.23). By contrast, Swiss and Polish breeds (ii) diverge in allometry from all other breeds and fowl (i + iii) (*F* = 2.58, *p* = 0.001) by showing a longer trajectory (*Z* = 2.9, *p *= 0.01) but not a different slope (*Z* = 0.075, *p* = 0.43). Given a shared common slope between the two groups (ii and i + iii), a test of LS means revealed a difference in intercept (*Z* = 11.2, *p *= 0.001).

### 3.3. Modularity and magnitude of variation in neural crest- and mesoderm-derived partsof the skull

The results of the modularity analysis do not support any of the given hypotheses (table 4). Strong integration among all landmarks is supported rather than a modular structure (table 4).

**Table 4.** Results of measures for modularity and integration. Measures of modularity and integration are based on CR (covariance ratio) and r-PLS (correlation score of partial least squares), respectively. In parentheses, *p*-values from 1000 random permutations. Denotations of Noden and Couly follow table 3.

	modularity		integration	
	*H*_Couly_	*H*_Noden_	*H*_Couly_	*H*_Noden_
alternative 1	1.002 (0.25)	0.941 (0.004)	0.952 (0.001)	0.925 (0.001)
alternative 2	0.984 (0.12)	0.968 (0.037)	0.946 (0.001)	0.943 (0.001)
alternative 3	0.986 (0.15)	0.975 (0.048)	0.941 (0.001)	0.954 (0.001)

The magnitude of variation (a) per hypothesized partition (as defined in table 3) and (b) per landmark reveals that (a) PV values for the neural crest partition *sensu* Couly *et al*. [40] are around twice (2.4–4.5) the magnitude of those for the *sensu* Noden [44] partition, after correction for differing numbers of landmarks per partition (table 5) and (b) landmarks that consistently contribute most to shape variation were placed at the cranial vault (containing the boundary of neural crest and mesoderm [44], or neural crest entirely [40]) (table 6).

**Table 5.** Results of magnitude of variation per hypothesized module in chickens following the definitions in table 3. Overall Procrustes variance (PV) for chickens is 0.017. PV is reported as calculated for each module and as standardized by the number of landmarks in each module (PV correct.).

		*H*_Couly_	*H*_Couly_	*H*_Noden_	*H*_Noden_
	module	PV	PV correct	PV	PV correct
alternative 1	neural crest	0.015 (75% LM = 90% of variation)	0.00083	46% LM = 71% of variation (PV = 0.012)	0.00109
	mesoderm	0.002 (25% LM = 10% of variation)	0.00033	54% = LM 28% of variation (PV = 0.005)	0.00039
alternative 2	neural crest	0.015 (63% LM = 86% of variation)	0.001	50% = LM 78% of variation (PV = 0.012)	0.001
	mesoderm	0.002 (37% LM = 14% of variation)	0.00022	50% LM = 22% of variation (PV = 0.005)	0.00042
alternative 3	neural crest	0.015 (70% LM = 89% of variation)	0.00088	58% LM = 80% of variation (PV = 0.014)	0.001
	mesoderm	0.002 (30% LM = 11% of variation)	0.00029	42% LM = 20% of variation (PV = 0.003)	0.0003

**Table 6.** Maximum Euclidean distance from the average configuration for each landmark and PV at each landmark, across all chicken specimens. Bold values are the two most variable midline and pairs of landmarks, which are also mentioned in the main text.

landmark no.	max Euclidean distance to mean configuration	PV
1	0.03764	**1**.**25058**
2, 3	0.02525	0.00043
4, 5	**0**.**06488**	0.000417
6, 7	0.02524	0.000223
8, 9	**0**.**13102**	**0**.**001059**
10, 11	0.04105	**0**.**001024**
12	**0**.**06357**	0.114994
13, 14	0.03665	0.000565
15	**0**.**05309**	0.234101
16	0.04095	**0**.**428121**
17	0.02549	0.419890
18, 19	0.03333	0.000597
20, 21	0.03101	0.000343
22, 23	0.02978	0.000423
24	0.02009	0.332442

## 4. Discussion

(Q1) The domesticated fowl,the chicken, in its different forms, occupies a larger portion of shape space than the wild form. (Q2 + 3) Trajectory analyses confirm a common allometric slope for domesticated and wild forms. Previous studies on bird skulls have found high levels of integration, and shape to be either controlled by ecological [51] or by developmental factors [4], but in all cases a strong shape–size correlation was reported. Our study on a domesticated species supports the role of high levels of cranial integration in birds but recovers only a weak magnitude of shape–size correlation. The latter result was probably impacted by domestication because the role of size variation in the evolutionary history of domesticated species has been shown to vary for mammals [52]. Here, selection for yield in meat-producing forms and reduced constraint on body size associated with lower predation may have played a role, although many co-occurring factors that induce variation in body size (e.g. extent of a species' geographical range in the case of some domesticates) may confound this signal (see [53]). We note that our sampling, although not exhaustive for breeds of chicken, considers the major categories accepted in both Europe and USA and captures most of the variation observed in chickens. Specimens include representatives of the Polish breed that nowadays shows an exaggerated enlargement of their crest due to over-selection of this trait for exhibition contests. Other peculiar kinds are the Araucana, as well as the Appenzeller Spitzhaubenhuhn—only preserved in one region of Switzerland—that shows cranial morphological similarities with crested breeds. Sampling of the RJF captures variation of the wild form, including individuals from three sources. Given a broad coverage of variation and selective regimes in our sample, our result appears to indicate that domestication may have altered the magnitude of the relationship between skull shape and size, as compared to the strong allometric signal that has been demonstrated for birds [49,54]. A previous comparison of craniofacial shape in domesticated birds, represented by pigeon breeds and avians has demonstrated that changes in trait covariance magnitudes (but not mode, i.e. patterning) occurred with domestication [4]. That is, domesticates and avians were shown to share a common pattern, but different amount of integration and allometric variation, accounting for diversification in craniofacial shape. Our allometric results provide further support for the role of magnitude, rather than mode, changes for trait covariances in generating variation on the short time scales associated with domestication, consistent with conclusions that have been drawn on the role of cranial integration magnitudes in generating diversity in mammals [12,55,56], and experimental evidence that directional selection can alter integration magnitudes [57,58]. Nevertheless, the extent to which changes to craniofacial integration and allometric variation may be generalized for different species brought into domestication requires further investigation. Particularly, recent evidence available for mammals suggests a species' response to domestication is highly variable, in the context of trait interactions, and may be related to size-variation associated with its evolutionary history [52] as well as the extent of trait maturity present at birth [59].

(Q4) The most variable portion of the chicken skull is the cranial vault (parietal, frontal), its embryonic origin currently disputed [39]. We have summarized additional skeletal cranial differences, which add to the here confirmed major differences between fowl and chickens and within chickens as revealed by PCA, in table 7. The difference in brain case shape between fowl and chickens and the variation in domesticated chicken breeds can be explained by the huge variation in brain shape and its reformation processes during ontogeny [60–62]. Regarding the variation in the upper premaxillary and nasal processes, we have no knowledge on the prevalence of the ‘gap' in the upper beak in other crested breeds. However, our sample also contained one crested Araucana skull, which did not exhibit this peculiarity, instead showing a protuberance in the cranial vault similar to the Polish skull. We conclude that the crested breeds exhibit skeletal abnormalities, expressed as either protuberances in the cranial vault or reduced premaxillary and nasal processes.

**Table 7.** Description of skeletal differences of the skull between fowls and chicken and within the analysed chicken breeds grouped by their breeding purposes.

type										
		chickens								
		ornamental breeds				laying breeds				
trait	fowls	fight	true bantams	other ornamental	Polish	Araucana	Appenzeller Barthuhn	Appenzeller Spitzhaubenhuhn	other laying	meat breed
premaxilla	straight	bended	slightly bended	bended	bended	bended	highly bended	bended	bended	bended
	In S/1985.41.2 (*Gallus sonneratii*) is bended like in chickens		In 63 (Holländisches Seidenhuhn) is straight as in fowls		In 1868.2. 19.61 (gold spangled Polish) beak is deformed					
cranial vault	Rounded/globular	Slight curvature in the boundary between frontal and parietal	Quite rounded/globular	Quite rounded/globular. Slightly furrowed frontal in the midline	Characteristic crest in the frontal. Curvature in the boundary between frontal and parietal is pronounced	Curvature in the boundary between frontal and parietal. Furrowed frontal in the midline	Slightly curvature in the boundary between frontal and parietal	Curvature in the boundary between frontal and parietal. Furrowed frontal in the midline with hole/holes between the frontals. The mesethmoid is quite visible between the frontal. A little ‘crest’ is present	Perceptible curvature in the boundary between frontal and parietal	Curvature in the boundary between frontal and parietal. Furrowed frontal in the midline
	1945.1.1 (*Gallus lafayetii*) shows two bilateral protuberances/knobs in the posterior part of the frontal		In 64 (Pekin) slightly furrowed frontal in the midline		In S/1952.2.114 (white Polish) the crest extends more anteriorly than in 1868.2.19.61 (gold spangled Polish)	In 115 there is a crest as in Polish but smaller, being the boundary between frontal and parietal even more pronounced. In 87 a little protuberance in the midline of the anterior part of the frontal			In s/1952.2.105 (Spanish cock) a little protuberance/ crest	In 2 (Swiss chicken) is quite rounded
nasals	Normally a perceptible suture with the frontal. In some specimens the nasals overlap the posterior-dorsal premaxila processes dorsally	Less perceptible suture with the frontal	Perceptible suture with the frontal, remarkable in 63 (Holländisches Seidenhuhn)	Perceptible suture with the frontal	Perceptible suture with the frontal and enlarged posteriorly. The anterior dorsal process does not meet the premaxilla	Fused with the frontal (suture imperceptible)	Perceptible suture with the frontal, showing a ‘V’ shape in this zone	Perceptible suture with the frontal and enlarged posteriorly The anterior dorsal process does not meet the premaxilla	Less perceptible suture with the frontal	Perceptible suture with the frontal
					In S/1952.2.114 (white Polish) meeting in the midline	In 115 more perceptible the suture with frontal, and enlargement to posterior		In 6 and 7 the suture with the frontal is less perceptible, in 2 nasals meet in the midline	In 7 (Leghorn) anterior dorsal processes overlap premaxilla	In S/1952.3.51 (Kulm-Malay) fused with the frontal (suture imperceptible)
supraoccipital/ foramen magnum	The posterior occipital end is rounded. Outline between supraoccipital and parietal rounded. Foramen magnum oriented to caudal	The posterior occipital end is slightly pointy. Outline between supraoccipital and parietal rounded. Foramen magnum oriented to ventral in 1868.2.1965 (Game hen) and caudal in 1868.2.19.58 (Rumpless fowl)	The posterior occipital end is pointy. Outline supraoccipital and parietal pointy (triangular). Foramen magnum oriented to caudal	The posterior occipital end is pointy. Outline supraoccipital and parietal rounded. Foramen magnum oriented to caudal	The posterior occipital end is rounded. Outline between supraoccipital and parietal pointy (triangular). Foramen magnum oriented to caudal	The posterior occipital end is pointy in 115 and 87, and quite rounded in 84 and 86. Outline between supraoccipital and parietal is rounded 84 and 86, quite straight in 115, and pointy in 87 (triangular). Foramen magnum oriented to caudal in 84 and 86, and ventral in 115 and 87	The posterior occipital end is rounded. Outline between supraoccipital and parietal rounded. Foramen magnum oriented to ventral (1 and 2) and caudal (3 and 4)	The posterior occipital end is pointy. Outline supraoccipital and parietal rounded. Foramen magnum oriented to caudal	The posterior occipital end is pointy. Outline between supraoccipital and parietal quite rounded. Foramen magnum oriented to caudal	The posterior occipital end is pointy. Outline between supraoccipital and parietal pointy (triangular). Foramen magnum oriented to ventral
	In S/1985.41.2 (*Gallus sonneratii*) the posterior occipital end is quite pointy		In 1868.2.19.74 (Burmese) outline between supraoccipital and parietal straight and foramen magnum oriented to ventral					In 6 and 7 the outline between supraoccipital and parietal pointy	In 7 (Leghorn) and S/1952.2.105 (Spanish cock) outline between supraoccipital and parietal is pointy (triangular). Foramen magnum oriented to ventral	
zygomatic/ postorbital process	meeting	meeting	meeting	meeting	not meeting	meeting	meeting	not meeting	meeting	meeting
								In 6 and 7 are meeting		

## 5. Conclusion

We found that the skull of the domesticated fowl, i.e. chickens, shows greater morphological variation than that of the wild fowl, a result that is consistent with those of many other studies that quantified shape variation of ancestral and domesticated breeds, like horses, pigs or pigeons. Bantams are closest to fowl in size–shape space, whereas Swiss breeds and the Polish deviate from the common shape–size trajectory of fowl and chickens by having a different intercept, irrespective of breed type (e.g. meat, egg, fight types). The observed variation is concentrated in the cranial vault, with neural crest-derived bones exhibiting higher amounts of variation; but variation can neither be attributed to changes in size (static allometry) nor to modularization. Instead, the chicken skull is strongly integrated. Cranial vault variation is rather attributable to brain shape variation, its extreme form expressed in crested breeds, such as the Polish.

## Supplementary Material

Supporting Figure S1

## Supplementary Material

Matrix of raw landmark data analysed in this study.

## Supplementary Material

R script 1. R code used to create Fig. 1 and Fig. S1.

## Supplementary Material

R script 2. R code used to conduct disparity analyses.

## Supplementary Material

R script 3. R code used to conduct modularity and integration analyses.

## Supplementary Material

Input.xlsx

## Data accessibility

Raw data and R code supporting this article have been uploaded as part of the electronic supplementary material.
