# Supplementary material for: Morphological variation under domestication: how variable are chickens?
Source: R Soc Open Sci. 2018 Aug 8;5(8):180993. doi: 10.1098/rsos.180993 (PMC6124038; doi:10.1098/rsos.180993)
Supplement: Supporting Figure S1 [file rsos180993supp1.docx]

Supporting Information

# Morphological variation under domestication: how variable are chickens?

Authors: Madlen Stange^1*^, Daniel Núñez-León^1^, Marcelo R. Sánchez-Villagra^1^, Per Jensen^2^, Laura A. B. Wilson^3*^

Addresses:

1. Paläontologisches Institut und Museum, Universität Zürich, Karl Schmid-Strasse 4, CH-8006 Zürich

2. AVIAN Behavioural Genomics and Physiology Group, IFM Biology, Linköping

University, 581 83 Linköping, Sweden

3. Palaeontology, Geobiology & Earth Archives Research Centre, School of Biological, Earth and Environmental Sciences, University of New South Wales, Sydney, NSW 2052

*Corresponding authors: stange.madlen@gmail.com, laura.wilson@unsw.edu.au

**Contents**

Figure S1. Scatterplot of the first two principal components from shape variables of fowl and chickens, without crested chicken breeds (Polish and Appenzeller Spitzhaubenhuhn).


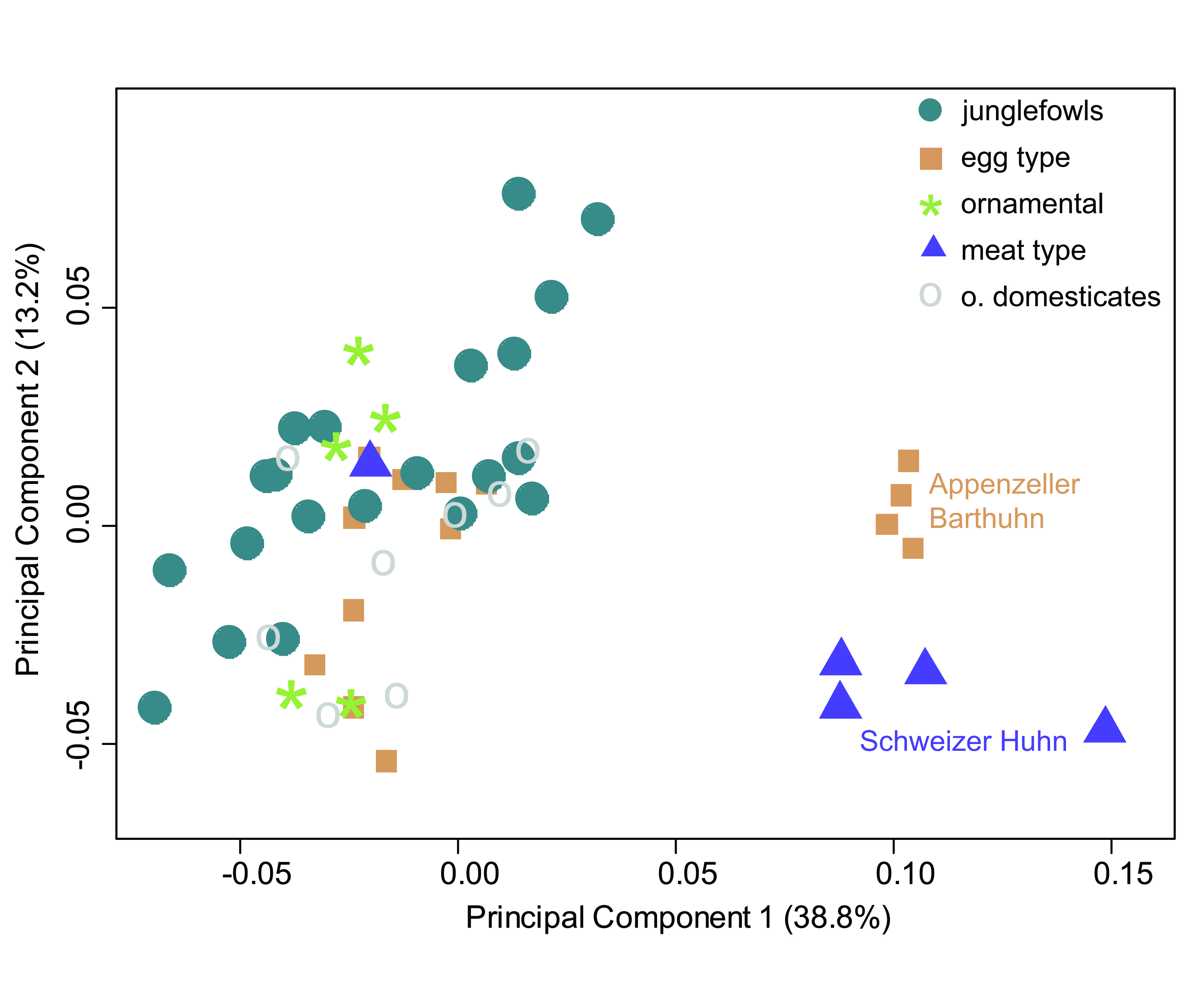


Figure S1. Scatterplot of the first two principal components from shape variables of fowl and chickens without crested chicken breeds (Polish and Appenzeller Spitzhaubenhuhn). The rare Swiss breeds are increasing variance along PC1, which is characterised by a more slender and more elongated and downward-curved skull in Swiss breeds (PC1 max) compared to the remaining tested breeds and fowl (PC1 min). Legend: green circles: fowl and crossbreeds; light green stars: ornamental breeds; orange squares: egg breeds; purple triangles: meat breeds; grey open circles: other domesticates with unknown breeding purpose.
